# Psychiatric morbidity is associated with cognitive impairment, whereas diagnostic category may shape its profile: a transdiagnostic study of inflammation, salivary biomarkers, and health-related quality of life

**DOI:** 10.3389/fpsyg.2026.1913919

**Published:** 2026-10-01

**Authors:** Andrea Pérez-Rivas, Marta Iglesias Martínez-Almeida, José Aguayo Arjona, María Sánchez Luaces, Juan Carlos Rodríguez Bernárdez, Francisco Javier Vicente Alba, María Elena de las Heras Linero, Adolfo Piñón-Blanco, Tania Rivera-Baltanás, Mario Navarro-Bardon, Eduardo Abalde-García, María del Carmen Vallejo-Curto, Francisco Otero-Lamas, José Antonio Campos-Pérez, Marta López-García, José Manuel Olivares, Carlos Spuch

**Affiliations:** 1 Translational Neuroscience Group, Galicia Sur Health Research Institute (IIS Galicia Sur), SERGAS-UVIGO, Vigo, Spain; 2 Primary Care Addiction Research Network (RIAPAD), Barcelona, Spain; 3 Grup de recerca en Nutrició Funcional, Oxidació i Malalties Cardiovasculars (NFOC-Salut) - Universitat Rovira i Virgili (URV), Reus, Spain; 4 Statistics and Methodology Unit, Galicia Sur Health Research Institute (IIS Galicia Sur), SERGAS-UVIGO, Vigo, Spain; 5 AFAGA Alzheimer Centre, Vigo, Spain; 6 Psychiatry Service, Vigo, Spain; 7 Biomedical Research Center Network for Mental Health (CIBERSAM), Madrid, Spain

**Keywords:** cognitive impairment, health-related quality of life, inflammation, psychiatric morbidity, salivary biomarkers, transdiagnostic

## Abstract

**Background:**

Cognitive impairment is highly prevalent across psychiatric disorders, but it remains unclear how psychiatric morbidity and diagnostic category contribute to its presence and profile and whether inflammation and salivary synaptic proteins are associated with cognitive impairment across diagnoses. This transdiagnostic study integrated clinical, cognitive, inflammatory, and salivary biomarker data to examine these questions.

**Methods:**

Cross-sectional assessment of 131 participants (controls, *n* = 63; major depressive disorder, *n* = 20; schizophrenia, *n* = 20; substance use disorder, *n* = 28). Systemic inflammation was indexed by neutrophil-to-lymphocyte ratio (NLR). Salivary synaptophysin (SYP), synaptotagmin-1 (SYT1), and synapsin-II (SYN2) were quantified as exploratory biomarkers. Logistic, multinomial, and linear regression models were used to identify variables independently associated with cognitive impairment, subjective distress, and inflammation.

**Results:**

The presence of any psychiatric disorder was the strongest independent correlate of cognitive impairment (OR = 5.23, 95% CI 2.05–13.35). Exploratory diagnosis-specific analyses suggested heterogeneity in cognitive, inflammatory, and psychosocial profiles. NLR was associated with psychiatric morbidity but was not independently associated with cognitive impairment. SYP showed an exploratory association with cognitive impairment (OR = 0.993 per ng/mL increase; corresponding OR = 0.681 across the observed interquartile range), although this finding was attenuated in sensitivity analyses and should be interpreted cautiously. Subjective distress was not independently associated with diagnosis or biomarkers but was strongly associated with health-related quality of life (HRQoL) (SF-12 physical and mental components; Nagelkerke *R*^2^ = 0.734), indicating a distinct psychosocial dimension; however, this finding should be interpreted cautiously because of conceptual overlap between self-reported constructs.

**Limitations:**

Cross-sectional design precludes causal inference. Sample size limits statistical power for diagnostic subgroup comparisons. The neuropsychological assessor was not blinded to participants’ diagnostic status. Universal psychopharmacological treatment precludes disentangling illness from medication effects.

**Conclusion:**

Psychiatric morbidity was associated with the presence of cognitive impairment in this baseline cross-sectional analysis, whereas the diagnostic category may contribute to heterogeneity in the impairment profile. Systemic inflammation was associated with psychiatric morbidity but was not independently associated with cognitive impairment. Subjective distress was strongly associated with HRQoL and appeared largely independent of the biological markers examined. These findings support an integrative multilevel framework in which cognitive impairment and HRQoL represent complementary transdiagnostic dimensions. Routine cognitive screening across psychiatric settings may be warranted. The observed SYP and HRQoL associations should be considered hypothesis-generating and require longitudinal validation.

## 1 Introduction

Neurocognitive impairment is a prevalent and functionally limiting feature of psychiatric disorders and is associated with poorer long-term outcomes, health-related quality of life (HRQoL), and reduced functional recovery (Czerwińska and Pawłowski, 2020; Huang et al., 2020; Neu et al., 2019). In contemporary models, this cross-diagnostic prevalence is conceptualized as a transdiagnostic “Factor C,” a dimension of cognitive dysfunction that spans traditional diagnostic boundaries (Abramovitch et al., 2021). High rates of cognitive impairment have been reported early in the course of illness, affecting up to 88.3% of individuals with recent-onset psychosis and 61.6% of those with recent-onset depression (Reviriego et al., 2025; Stainton et al., 2023). In substance use disorders (SUD), the estimated global prevalence in treatment-seeking outpatients is approximately 31.0% (Bruijnen et al., 2019), though studies focusing on severe or active consumption patterns report rates as high as 74.9% (Reviriego et al., 2025). These deficits often persist even during periods of clinical remission, particularly in major depressive disorder (MDD), where they are linked to higher relapse risk and reduced treatment response (Czerwińska and Pawłowski, 2020; Neu et al., 2019). The persistence of these deficits across diagnostic categories and clinical states underscores their relevance as a transdiagnostic treatment target for mental health (Abramovitch et al., 2021; Stainton et al., 2023).

This perspective aligns with growing evidence that categorical diagnostic models have limitations, as they often fail to fully capture the shared biological mechanisms underlying various psychiatric disorders (Abramovitch et al., 2021). Neurobiological evidence supports this perspective: connectome studies have identified a common hyper-integrated network architecture, characterized by reduced modularity in frontoparietal and subcortical regions, across schizophrenia (SZ), bipolar disorder (BD), and MDD (Ma et al., 2020). These shared alterations in key hubs, such as the dorsolateral prefrontal cortex and thalamus, offer a plausible neurobiological substrate for transdiagnostic cognitive dysfunction (Abramovitch et al., 2021; Ma et al., 2020). Comparable cross-diagnostic commonalities extend to cognitive biases and structural brain changes, including reduced gray matter volume in the medial superior frontal cortex observed in both SZ and MDD (Ma et al., 2021). Moreover, genetic and epidemiological studies have identified major mental disorders as independent risk factors for Alzheimer’s disease, suggesting that transdiagnostic cognitive impairment may reflect a shared vulnerability to neurodegeneration in later life (Liu et al., 2024). Taken together, evidence spanning network architecture and genetic vulnerability supports the hypothesis that a shared neurobiological substrate may manifest clinically as transdiagnostic cognitive impairment and biologically as low-grade inflammation and potential synaptic dysregulation, which the present study aims to investigate.

In this context, systemic inflammation has emerged as a key transdiagnostic pathophysiological mechanism. Elevated peripheral inflammatory markers, such as the practical and low-cost neutrophil-to-lymphocyte ratio (NLR), are frequently reported in psychiatric disorders and are associated with poorer cognitive performance (Canli, 2024; Chen et al., 2025). Longitudinal evidence suggests that these markers predict cognitive decline and progression to amnestic states in psychiatric populations (Cervellati et al., 2024; Syahna et al., 2025). However, recent evidence also indicates that inflammation may interact with, rather than directly determine, cognitive outcomes, with its apparent effects diminishing when clinical or functional factors are considered. Longitudinal studies confirm that inflammatory-cognitive subgroups are stable over time and closely associated with clinical severity (Sæther et al., 2024).

Psychosocial and subjective health factors represent a distinct pathway, as evidenced in aging populations, where comprehensive health measures are linked to cognitive status, with depression acting as a key mediator of the relationship between physical health and impairment (Yates et al., 2017). This dual-pathway framework, biological and psychosocial, aligns with contemporary models of cognitive vulnerability in psychiatry.

Beyond neurocognitive impairment and systemic inflammation, subjective and functional dimensions, particularly HRQoL and perceived distress, have emerged as central components of clinical burden in psychiatry. These psychosocial constructs capture aspects of everyday functioning and subjective wellbeing that are not fully explained by symptom severity or diagnostic labels, and they may operate through pathways partially distinct from biological markers. However, the majority of studies have examined biological and psychosocial factors in isolation, limiting our understanding of how they jointly contribute to cognitive outcomes.

In parallel, the search for minimally invasive biomarkers has gained momentum, with saliva offering a practical and low-burden biofluid for translational research. Although salivary assays have been used to quantify cortisol, inflammatory indices, and even neuronally derived proteins in neurodegenerative conditions, their application to psychiatric populations remains limited. Integrating both dimensions, the present study utilizes a novel exploratory salivary panel to examine whether peripheral synaptic signals are associated with cognitive impairment and HRQoL. By simultaneously assessing biological (inflammation, exploratory synaptic proteins) and psychosocial (HRQoL, perceived stress) factors within a transdiagnostic framework, we aimed to determine their relative contributions to cognitive impairment and subjective distress.

The collective evidence suggests that cognitive impairment in psychiatry is a multidimensional phenomenon involving shared biological mechanisms, diagnosis-specific factors, and subjective psychosocial states. However, these levels are typically examined separately, limiting our understanding of their relative distribution and interactions. To address this gap, the present study employed a cross-sectional, transdiagnostic design integrating clinical, neuropsychological, and biomarker data with three primary objectives: (1) to quantify the association between psychiatric disorders (MDD, SZ, and SUD) and cognitive impairment and identify potential biological correlates; (2) to compare the cognitive, clinical, and biological characteristics of individuals with MCI across diagnoses; and (3) to examine the associations of an inflammatory marker (NLR), exploratory synaptic proteins, and psychosocial factors (HRQoL) with cognitive impairment and subjective distress.

## 2 Methods

### 2.1 Study design and participants

This cross-sectional study represents the baseline assessment of an ongoing prospective longitudinal cohort study. Participants were consecutively enrolled over 6 months (January–June 2024) from collaborating clinical centers in Vigo, Spain. Recruitment targeted four diagnostic groups: controls (C, *n* = 63), patients with MDD (*n* = 20), SZ (*n* = 20), and SUD (*n* = 28). Clinical diagnoses were established by treating psychiatrists according to the DSM-5 criteria and subsequently verified through clinical record review by a senior researcher. To ensure non-overlapping and analytically independent groupings, participants were later reclassified into mutually exclusive psychiatric × cognitive groups, consistent with the study’s transdiagnostic framework.

### 2.2 Sample size justification and characteristics

An *a priori* power analysis (G*Power 3.1.9.7) was conducted for the most demanding planned comparison: a one-way ANOVA across five core analytical groups (see section 2.6 for group derivation): C without impairment, a pooled group of patients with psychiatric disorders but no cognitive impairment, and diagnosis-specific groups of patients with MDD, SZ, or SUD with comorbid MCI. Assuming a medium effect size (*f* = 0.30), α = 0.05, and power = 0.80, the analysis indicated a required sample size of 140 participants (28 per group).

The final sample comprised 131 participants. Although slightly below target, this sample size provided adequate power (>0.75) for the primary transdiagnostic objective of quantifying psychiatric disorder status as a factor associated with cognitive impairment, which was the central aim of the cross-sectional baseline assessment. The power calculation was based on the originally planned analytical grouping strategy. Following data inspection and the hierarchical analytical approach ultimately adopted, some exploratory subgroup analyses were restructured to address specific study objectives more appropriately. Therefore, the Level 2 and Level 3 groupings should not be interpreted as a direct one-to-one implementation of the original power-calculation grouping scheme.

Group size imbalance, particularly the larger control group, reflects the challenges of recruiting clinically stable psychiatric participants for biomarker-oriented, multi-level studies. This imbalance moderately reduces statistical power for diagnosis-specific comparisons but does not compromise the primary transdiagnostic analyses. Accordingly, Level-3 (diagnosis-specific) findings are considered exploratory and hypothesis-generating, requiring replication in larger, balanced cohorts. The longitudinal structure of the cohort will additionally provide repeated measures over the years to improve precision and enable assessment of temporal relationships.

### 2.3 Eligibility criteria and clinical definitions

- Inclusion Criteria: All participants were required to be aged >18 years and fluent in Spanish. For patient groups, a confirmed DSM-5 diagnosis of MDD, SZ, or SUD was required. All participants had to sign a written informed consent form in accordance with the Declaration of Helsinki, as this study had been approved by the Pontevedra-Vigo-Ourense Ethics Committee (Ref: 2024/427).
- Exclusion Criteria: History of neurological disorder (e.g., dementia, epilepsy, traumatic brain injury with loss of consciousness >30 min), intellectual disability, or active medical illness affecting CNS function (Table 1).

**Table 1:** Inclusion criteria based on cognitive status and clinical stability.

Criteria	Controls	Experimental groups (MDD, SZ, SUD)
Subjective cognitive complaints	None reported.	Subjective complaints (patient or clinician).
Cognitive performance	Performance within ± 1.0 SD on all primary cognitive domain tests.	Score below the adjusted cut-off on MoCA (Spanish Normacog). MCI: ≥ 1.0 SD below norms on ≥ 2 of 3 primary tests. ModCI: ≥ 1.5 SD below norms on ≥ 2 primary tests. SevCI: ≥ 2.0 SD below norms on ≥ 2 primary tests.
Clinical stability	No psychiatric diagnosis required.	MDD: No major depressive episode in the prior 4 weeks. SZ: ≥ 1 month post-acute episode. SUD: Abstinence ≥ 4 weeks (verified by toxicology).
Medication	No psychotropic medication.	Stable psychopharmacological regimen for ≥ 4 weeks

Primary test: HVLT-R Total Recall, Stroop Color-Word, Digit Span Backward. Severe impairment (SevCI) reported descriptively only.

### 2.4 Neuropsychological assessment

Cognitive function was assessed using a standardized battery administered by a trained neuropsychologist. Global cognition was evaluated using the Montreal Cognitive Assessment (MoCA), with Spanish Normacog-adjusted norms. Verbal learning and memory were assessed using the Hopkins Verbal Learning Test-Revised (HVLT-R), assessing immediate recall, total learning over three trials, delayed recall, and recognition discriminability. Executive function and processing speed were assessed using the Stroop Color-Word Test (Golden Version), yielding word reading and color-word interference scores. Working memory was examined using the Digit Span subtest (forward and backward) from the WAIS-III. All raw scores were converted to demographically adjusted z-scores using published Spanish normative data (NEURONORMA Project).

In addition, participants completed the Cognitive Reserve Questionnaire (CRC), the Hospital Anxiety and Depression Scale (HADS), the Perceived Stress Scale (PSS), and the SF-12 Health Survey, which provides physical (PCS) and mental (MCS) component summaries. Cognitive impairment was classified according to performance relative to normative data: mild cognitive impairment (MCI) was defined as performance ≥1.0 standard deviation (SD) below norms on at least two primary tests (HVLT-R total recall, Stroop Color-Word, Digit Span backward), moderate impairment (ModCI) as ≥1.5 SD below norms on at least two primary tests, and severe impairment (SevCI) as ≥2.0 SD below norms on at least two primary tests (Table 2).

**Table 2:** Neuropsychological assessment battery.

Cognitive domain	Test/instrument	Normative data (Spanish)
Global cognition	Montreal Cognitive Assessment (MoCA)	Spanish Normacog Version (Ojeda et al., 2016)
Working memory	Digit Span (Forward/Backward; WAIS-III)	NEURONORMA Project (Peña-Casanova et al., 2009b; Tamayo et al., 2012)
Verbal learning and memory	Hopkins Verbal Learning Test-Revised (HVLT-R)	NEURONORMA project (Sáez-Atxukarro et al., 2021)
Executive function/processing speed	Stroop Color-Word Test (Golden Version)	Peña-Casanova et al. (2009a) and Rognoni et al. (2013)
Self-report measures	Cognitive Reserve Questionnaire (CRC)	Martino et al. (2022)
	Hospital Anxiety and Depression Scale (HADS)	Terol et al. (2007)
	Perceived Stress Scale (PSS)	Remor (2006)
	SF-12 Health Survey (PCS, MCS)	Schmidt et al. (2012)

The battery was administered by a trained neuropsychologist (A. P. R.) in a standardized order within a controlled environment. Raw scores were converted to demographically adjusted z-scores using the corresponding published Spanish normative datasets to allow for cross-domain comparison. Although the assessor was not blinded to diagnostic status, strict adherence to standardized procedures and the use of norm-referenced scoring minimized potential subjective influence.

For the primary regression analyses, cognitive impairment was dichotomized as impaired versus non-impaired to evaluate the main transdiagnostic question with adequate statistical stability. One participant did not complete the MoCA assessment and could not be classified for cognitive impairment status. Consequently, analyses involving cognitive impairment categories were conducted on 130 participants. Severity-specific categories were retained for descriptive and exploratory analyses because separate modeling of MCI, moderate impairment, and severe impairment would have produced sparse cells in the available sample.

### 2.5 Biomarker assessment

The NLR and Lymphocyte-to-Monocyte Ratio (LMR) were calculated from differential white blood cell counts obtained from the participants’ most recent routine clinical blood counts, consistent with practical clinical biomarker approaches used in psychiatric inflammation research. The use of routine data captures clinically meaningful inflammatory status but introduces unavoidable temporal variability, which is acknowledged in the interpretation.

For salivary analyses, unstimulated whole saliva was collected immediately after neuropsychological testing following 1 h of abstinence from caffeine, tobacco, food, and exercise. Participants rinsed their mouths with water and passively drooled for 2 min into pre-chilled tubes. Samples were promptly centrifuged at 3,000 × g for 10 min at 4 °C, aliquoted, and stored at −80 °C until analysis.

Cortisol was quantified in duplicate using a commercial ELISA kit (ABclonal, RK00639) according to the manufacturer’s instructions. Similarly, concentrations of SYP (Human SYP, MyBioSource, MBS045528), SYT1 (Human SYT1, MyBioSource, MBS9312725), and SYN2 (Human SYN2, MyBioSource, MBS9312722) were quantified in duplicate using commercial ELISA kits and normalized to total salivary protein determined using a bicinchoninic acid (BCA) assay (Pierce™ BCA Protein Assay Kit). Absorbance was read on a microplate reader (Omega Mars, BMG Labtech).

### 2.6 Statistical analysis

Analyses were performed using IBM SPSS Statistics v29.0 software (IBM Corp., Armonk, NY, United States). Significance was set at α = 0.05 (two-tailed). Continuous variables were assessed for normality using the Kolmogorov–Smirnov test and are presented as median (interquartile range, IQR) when they were non-normally distributed; categorical variables are presented as frequencies and percentages (n, %).

Regression models used a complete-case analysis according to the variables entered in each model. Univariate comparisons across Levels 1–3, biomarker comparisons, and sex-difference analyses are therefore reported as exploratory and hypothesis-generating, with unadjusted overall *p*-values shown for transparency. For Level 2 pairwise post-hoc comparisons, Benjamini–Hochberg-adjusted *p*-values were used to control the false discovery rate.

Sensitivity analyses were conducted for Model 1 and are reported in Supplementary Table S1. These included (a) a model using all participants with available SYP data, (b) a core sociodemographic-adjusted model including age, sex, education, socioeconomic status, and family support, and (c) an expanded model additionally including cognitive reserve, anxiety, depression, and SF-12 physical and mental component scores. Biomarker missingness was examined according to cognitive impairment status, psychiatric morbidity, and diagnostic group and is summarized in Supplementary Table S2. Sensitivity analyses additionally included age and years of education as covariates because both variables differed across diagnostic groups. Neuropsychological outcomes and cognitive impairment classifications were based on demographically adjusted Spanish normative data, thereby incorporating age- and education-related corrections into the primary cognitive classification.

We employed a hierarchical analytical approach with three complementary levels based on non-redundant group classifications:

- Level 1: Cognitive Impairment Status: Participants were dichotomized as cognitively impaired (CI+, *n* = 68) or cognitively non-impaired (CI−, *n* = 62). Group comparisons were performed using Mann–Whitney U tests (continuous variables) and Chi-square (χ^2^) tests (categorical variables). This dichotomous classification was selected for the primary analyses because the principal study objective was to evaluate the transdiagnostic association between psychiatric morbidity and the presence of cognitive impairment. Severity-specific analyses were considered exploratory and were evaluated separately in the subsequent analytical levels.
- Level 2: Combined psychiatric × cognitive groups: To explore clinically relevant combinations of cognitive impairment severity and psychiatric morbidity, a subset of participants was categorized into four selected mutually exclusive exploratory strata: no impairment/no disorder (MCI-TM-, *n* = 46), MCI without active psychiatric disorder (MCI + TM-, *n* = 17), MCI with a comorbid psychiatric disorder (MCI + TM+, *n* = 27), and moderate cognitive impairment with a comorbid psychiatric disorder (ModCI+TM+, *n* = 13). Participants with psychiatric disorders but without cognitive impairment (*n* = 21), as well as the small number of participants with severe cognitive impairment (*n* = 6), were not included in this exploratory stratified analysis. Therefore, this classification was not intended to provide an exhaustive representation of the full cohort. Group comparisons were performed using Kruskal–Wallis H tests (continuous) and chi-square (χ^2^) tests (categorical).
- Level 3: Diagnostic Specificity within MCI: Analyses focused on MCI cases (*n* = 45): MCI-only (*n* = 18), SZ + MCI (*n* = 10), MDD + MCI (*n* = 7), and SUD + MCI (*n* = 10). Given the small and unbalanced samples, these analyses were considered exploratory and hypothesis-generating.

Regression Models: To test primary hypotheses while controlling confounders, we specified several regression models. Model fitting used backward stepwise elimination (removal *p* > 0.10). Models were checked post-estimation for assumptions and multicollinearity and simplified accordingly. The following were tested:

- Model 1. Factors Associated with Cognitive Impairment: Binomial logistic regression was used to identify variables independently associated with cognitive impairment, including psychiatric disorder status, cognitive reserve, psychosocial factors, inflammatory markers, and salivary synaptic proteins.
- Model 2. Factors Associated with Subjective Distress: Multinomial logistic regression identified SF-12 physical and mental components as the only variables independently associated with subjective distress.
- Model 3. Factors Associated with Systemic Inflammation: Linear regression identified psychiatric disorder status as the only variable independently associated with NLR.

Given the large number of comparisons and small subgroup sizes, diagnosis-specific findings were interpreted cautiously and reported as exploratory. Sex differences were examined using Mann–Whitney U tests. Bivariate relationships were explored using Spearman’s ρ correlations.

## 3 Results

Analyses were conducted following a hierarchical, conceptually driven strategy. First, global cognitive impairment status was examined across the full sample (Level 1). Second, the interaction between cognitive status and psychiatric morbidity was explored to reflect clinical complexity (Level 2). Finally, diagnosis-specific profiles within mild cognitive impairment were examined as exploratory analyses (Level 3), given sample size limitations.

### 3.1 Sample characteristics

The final sample comprised 131 participants. One participant did not complete the MoCA assessment and could not be classified for cognitive impairment status; therefore, analyses involving cognitive impairment status were conducted on 130 participants. The median age was 45 years, 52.7% were women, and 58.8% had higher education. Psychiatric diagnoses were distributed as follows: MDD (*n* = 20), SZ (*n* = 20), and SUD (*n* = 28). Cognitive impairment (MCI or higher) was present in 68 participants, representing 52.3% of the sample (Tables 3–6).

**Table 3:** Sociodemographic and general clinical characteristics of the total sample (*N* = 131).

Characteristic	Category	Total *n* (%)/median [IQR]
Sociodemographic		
Age (years)		45 [30–57]
Sex	Male	62 (47.3)
	Female	69 (52.7)
Educational level	Primary or lower	22 (16.8)
	Secondary	32 (24.4)
	Higher	77 (58.8)
Years of education		14 [10–17]
Marital status	With partner	56 (42.7)
	Without partner	75 (57.3)
Living environment	Urban	121 (92.4)
	Rural	10 (7.6)
Socioeconomic level	Low	72 (55.0)
	Medium	56 (42.7)
	High	3 (2.3)
Family support	Low	27 (20.6)
	Medium	37 (28.2)
	High	67 (51.1)
Employment status	Student	10 (7.6)
	Employed	43 (32.8)
	Unemployed/on medical leave	36 (27.5)
	Retired/pensioner	42 (32.1)
Family history (first-degree relatives)		
Dementia		23 (17.6)
Schizophrenia		6 (4.6)
Bipolar disorder		6 (4.6)
Major depressive disorder		38 (29.0)
Substance use disorder		27 (20.6)
Other		16 (12.2)
Past clinical history		
Past psychiatric diagnosis	Yes	60 (45.8)
	No	71 (54.2)
Past neurological condition	Yes	9 (6.9)
	No	122 (93.1)
Previous hospitalizations	Yes	36 (27.5)
	No	95 (72.5)
Number of previous hospitalizations*		2 [1–5]
Suicidal ideation (history)	Yes	46 (35.1)
	No	85 (64.9)
Suicide attempts (history)	Yes	19 (14.5)
	No	112 (85.5)
Self-harm (non-suicidal)	Yes	12 (9.2)
	No	119 (90.8)

Data are presented as median (IQR) for continuous variables and n (%) for categorical variables. *Calculated only among participants with a history of psychiatric hospitalization (*n* = 36).

**Table 4:** Current clinical, emotional, and treatment characteristics of the total sample (*N* = 131).

Characteristic	Category/measure	Total
Substance use		
Substance use disorder (lifetime)	Yes	51 (38.9%)
	No	80 (61.1%)
Current substance use (past 30 days)	Yes	43 (32.8%)
	No	88 (67.2%)
Specific substances used		
Tobacco		43 (32.8%)
Alcohol		5 (3.8%)
Cannabis		13 (9.9%)
Cocaine		5 (3.8%)
Opioids		0 (0%)
Current treatment		
Psychopharmacological treatment*	Yes	89 (67.9%)
	No	42 (32.1%)
Psychotropic medication classes (total sample)		
Antidepressants		59 (45.0%)
Anxiolytics/hypnotics		56 (42.7%)
Antipsychotics		39 (29.8%)
Emotional and subjective health status		
Depression (HADS)	Normal	91 (69.5%)
	Possible case	18 (13.7%)
	Case	22 (16.8%)
Anxiety (HADS)	Normal	56 (42.7%)
	Possible case	28 (21.4%)
	Case	47 (35.9%)
Perceived stress (PSS)	Normal	26 (19.8%)
	Mild	53 (40.5%)
	Moderate	42 (32.1%)
	Severe	10 (7.6%)
Health-related quality of life (SF-12)		
Physical component summary (PCS)		54.1 [43.2–57.3]
Mental component summary (MCS)		42.3 [26.7–62.4]

Data are presented as median (IQR) for SF-12 scores and n (%) for categorical variables. *Detailed psychopharmacological profiles appear in Table 1.

**Table 5:** Cognitive characteristics and neuropsychological test performance of the total sample (*N* = 130).

Characteristic	Category/domain	Total
Cognitive classification		
Cognitive status	No cognitive impairment (no MCI)	62 (47.3%)
	MCI	45 (34.4%)
	ModCI	17 (13.0%)
	SevCI*	6 (4.6%)
Cognitive reserve		
Cognitive reserve questionnaire (CRC)	Inferior	13 (9.9%)
	Low	1 (0.8%)
	Low-middle	22 (16.8%)
	Middle-high	36 (27.5%)
	Superior	59 (45.1%)
Neuropsychological tests		
Global cognition		
MoCA—total score		27 [24–29]
Verbal memory (HVLT-R)		
Trial 1		9 [6–11]
Total recall (trials 1–3)		9 [6.5–11]
Delayed recall (trial 4)		11 [7–15]
Recognition discriminability		16 [11–17]
Executive function/processing speed (Stroop test)		
Word reading		11 [8–13]
Color-word interference		10 [8–13]
Working memory (digit span)		
Forward		10 [9–13]
Backward		11 [9–13]

One participant with an incomplete MoCA was excluded. Data are presented as median [IQR] for test scores and n (%) for categorical variables. *SevCI refers to ≥2 SD impairment on ≥2 tests.

**Table 6:** Inflammatory, hormonal, and synaptic profile of the study population (*N* = 131).

Variable	*n*	Median (IQR)
Inflammatory markers		
NLR	106	1.47 [1.12–1.94]
LMR	106	5.10 [3.76–6.16]
Hormonal markers		
Basal TSH (μIU/mL)	65	2.36 [1.56–3.41]
Cortisol (ng/mL)	69	0.00 [0.00–1.40]
Salivary synaptic proteins		
SYP (ng/mL)	110	40.48 [17.42–66.62]
SYT1 (ng/mL)	129	0.36 [0.31–0.52]
SYN2 (ng/mL)	130	0.21 [0.15–0.30]

Data are presented as median (IQR). Variable sample sizes reflect insufficient saliva volume for duplicate ELISA analyses in some participants, commonly related to medication-induced xerostomia in psychiatric populations.

Age differed across diagnostic groups (Kruskal–Wallis *p* = 0.028), and years of education differed more markedly (*p* < 0.001), with participants in the SUD group having the lowest educational attainment (Supplementary Table S3).

Missing biomarker data did not differ according to cognitive impairment status. However, missingness differed across diagnostic groups for NLR and SYP and showed a trend according to psychiatric morbidity for SYP (Supplementary Table S2).

### 3.2 Global differences by cognitive impairment status

Participants with cognitive impairment (CI+; *n* = 68) showed significantly poorer performance across all cognitive domains compared with those without impairment (CI-; *n* = 62) (all *p* < 0.001). They also showed higher rates of clinically significant anxiety and depression (HADS; *p* = 0.002 for both). Regarding psychosocial factors, CI + participants showed lower mental HRQoL (SF-12 MCS; *p* = 0.006) and reported significantly lower perceived family support (*p* = 0.018) (Table 7).

**Table 7:** Comparison of characteristics between participants with (CI+) and without (CI−) cognitive impairment (*N* = 130).

Characteristic	CI−	CI+	*p*-value
Sociodemographic			
Family support			0.018^1^
—Low	6 (9.7)	20 (29.4)*	
—Medium	21 (33.9)	16 (23.5)	
—High	35 (56.5)	32 (47.1)	
Cognitive test performance			
MoCA total score	29 [27.8–30]	25 [21–27]	<0.001^2^
Verbal fluency (total words)	11 [9–13.3]	9 [7–11]	<0.001^2^
HVLT-R			
—Trial 1	9 [8–13]	8 [6–10]	0.003^2^
—Total recall (trials 1–3)	10 [8–13.3]	7 [4–10]	<0.001^2^
—Delayed recall (trial 4)	15 [10–16]	7.5 [4–12]	<0.001^2^
—Recognition discriminability	16 [15–17]	15 [8–17]	0.011^2^
Stroop test			
—Word	12 [10–14]	8.5 [6–12]	<0.001^2^
—Color-word	12 [10–14]	8.5 [6–11.3]	<0.001^2^
Digit span forward	10.5 [9–13.3]	10 [8–11]	0.004^2^
Digit span backward	12 [11–14]	10 [8–13]	<0.001^2^
Clinical characteristics			
Cognitive reserve (CRC)			0.038^1^
—Low	4 (6.5)	10 (14.7)	
—Low-middle	6 (9.7)	16 (23.5)*	
—Middle-high	21 (33.9)	14 (20.6)	
—Superior	31 (50.0)	28 (41.2)	
Depression (HADS)			0.002^1^
—Normal	52 (83.9)*	38 (55.9)	
—Possible case	4 (6.5)	14 (20.6)*	
—Case	6 (9.7)	16 (23.5)*	
Anxiety (HADS)			0.002^1^
—Normal	36 (58.1)*	19 (27.9)	
—Possible case	10 (16.1)	18 (26.5)	
—Case	16 (25.8)	31 (46.5)*	
Quality of life (SF-12)			
—Mental component	48.5 [30.1–53.5]	34.2 [22.4–50.3]	0.006^2^

One participant was excluded for missing MoCA. Data expressed as median [IQR] or n (%). Asterisks (*) denote significant post-hoc differences. ^1^Chi-square test; ^2^Mann–Whitney U test.

Inflammatory markers (NLR, LMR), cortisol, and exploratory synaptic proteins (SYP, SYT1, SYN2) did not differ significantly between CI + and CI- (all *p* > 0.05). No group differences emerged in socioeconomic status, subjective distress, or the SF-12 Physical Component (all *p* > 0.05) (Table 8).

**Table 8:** Comparison of sociodemographic, cognitive, and clinical characteristics between participants with and without cognitive impairment.

Biomarker	*p*-value
Inflammatory	
NLR	0.852
LMR	0.302
Hormonal	
Cortisol (ng/mL)	0.785

Synaptic proteins	*p*-value
SYP (ng/mL)	0.942
SYT1 (ng/mL)	0.232
SYN2 (ng/mL)	0.612
Total protein (ug/mL)	0.389

Mann–Whitney U tests for all comparisons (CI+ vs. CI−). No significant differences were observed (all *p* > 0.05).

### 3.3 Interplay between cognitive impairment and psychiatric status

To examine the combined effect of cognitive impairment severity and psychiatric morbidity, an exploratory Level 2 analysis was conducted using the four-group variable defined in the Methods. The distribution of cognitive impairment severity across diagnostic groups is shown in Supplementary Table S4.

When participants were stratified by both cognitive and psychiatric status (MCI-TM- [*n* = 46], MCI + TM- [*n* = 17], MCI + TM + [*n* = 27], ModCI+TM + [*n* = 13]), consistent group differences emerged (Table 9).

**Table 9:** Characteristics by combined cognitive and psychiatric status (*n* = 130).

Characteristic	MCI- TM-	MCI + TM-	MCI + TM+	ModCI+ TM+	*p*-value	*p*-value groups
Sociodemographic						
Socioeconomic level					0.005^1^	
—Low	39 (52.7)	6 (35.3)	18 (66.7)	9 (62.9)		
—Medium	34 (45.9)	11 (64.7)*^a^	9 (33.3)	2 (15.4)*^a^		0.005^1a^
—High	1 (1.4)*^a^	0 (0.0)	0 (0.0)	2 (15.4)*^a^		0.005^1a^
Cognitive test performance						
MoCA total score	29 [27–30]*^ab^	28 [24.5–28.5]*	24 [25–26]*^a^	21 [19–23]*^ab^	<0.001^2^	0.002^2b^/<0.001^2a^
Verbal fluency (total words)	11 [9–13]*^a^	10 [9–13]*^b^	10 [8–11]	5 [3.5–9.5]*^ab^	<0.001^2^	0.007^2b^/<0.001^2a^
HVLT-R						
Total recall (trials 1–3)	10 [7–12]*^a^	9 [7.5–10]*^b^	7.5 [6–10.5]	5 [2.5–7]*^ab^	<0.001^2^	0.027^2b^/<0.001^2a^
Delayed recall (trial 4)	12 [8.5–16]*^a^	10 [9–15.5]*^b^	7.5 [5.8–13]	4.5 [3–7.5]*^ab^	<0.001^2^	0.004^b^/<0.001^2a^
Recognition discriminability	16 [12.5–17]*^a^	16 [14.5–17]	15 [9.5–17]	10 [4.8–14]*^a^	0.027^2^	0.018^2a^
Stroop						
Word	12 [9–14]*^a^	12 [11–14]*^b^	8 [4.8–10.5]*^ab^	9 [6–10]	<0.001^2^	0.007^2b^/<0.001^2a^
Color-word	12 [9–14]*^a^	13 [9.5–14.5]*^ab^	8 [5.8–10]*^a^	6.5 [5.3–9.8]*^b^	<0.001^2^	0.002^2b^/<0.001^2a^
Digit span backward	12 [10–13.5]*^a^	13 [10–13.5]*^b^	10.5 [8–11.5]	9 [7.5–10.5]*^ab^	<0.001^2^	0.006^2b^/0.004^2a^
Clinical characteristics						
Cognitive reserve (CRC)					0.004^1^	
—Low	5 (6.8)*^a^	0 (0.0)	5 (18.5)	4 (30.8)*^a^		0.011^1a^
—Low-middle	10 (13.5)	3 (17.6)	7 (25.9)	2 (15.4)		
—Middle-high	25 (33.8)	1 (5.9)	6 (22.2)	4 (30.8)		
—Superior	34 (45.9)	13 (76.5)*^a^	9 (33.3)*^a^	3 (23.1)*^a^		0.011^1a^
Depression (HADS)					<0.001^1^	
—Normal	59 (79.7)*	15 (88.2)*	12 (44.4)*	5 (38.5)*		
—Possible case	7 (9.5)	2 (11.8)	7 (25.9)	2 (15.4)		
—Case	8 (10.8)*	0 (0.0)	8 (29.6)	6 (46.2)*		
Anxiety (HADS)					<0.001^1^	
—Normal	39 (52.7)*	9 (52.9)	5 (18.5)	3 (23.1)		
—Possible case	12 (16.2)	6 (35.3)	7 (25.9)	3 (23.1)		
—Case	23 (31.1)	2 (11.8)	15 (55.6)*	7 (53.8)		
Perceived stress (PSS)					<0.001^1^	
—Normal	17 (23.0)	6 (35.5)*^a^	1 (3.7)*^a^	2 (15.4)		0.012^1a^
—Mild	29 (39.2)	8 (47.1)	13 (48.1)	3 (23.1)		
—Moderate	23 (31.1)	3 (17.6)	12 (44.4)	4 (30.8)		
—Severe	5 (6.8)*^a^	0 (0.0)	1 (3.7)*^a^	4 (30.8)*^a^		0.012^1a^
Quality of life (SF-12)						
—Physical component	54 [47.7–57.3]	56.8 [44.8–59.5]*^a^	53 [42.2–57.8]	40.7 [30.3–54.8]*^a^	0.019^2^	0.014^2a^
—Mental component	47.6[29.2–53.4]*^b^	49.5 [34.4–53.8]*^ac^	30.5[21.3–42.8]*^ab^	27.5 [16.4–45.9]*^c^	<0.001^2^	0.035^2c^/0.031^2b^/0.024^2a^

Data are presented as n (%) or median [IQR]. Significant overall group differences were assessed using χ^2^ tests (1) or Kruskal–Wallis tests (2). Groups sharing the same superscript letter (a, b, or c) differ significantly from each other in pairwise *post-hoc* comparisons. Corresponding *post-hoc p*-values are reported in the final column. *p < 0.05

Groups with comorbid psychiatric disorders (MCI + TM + and ModCI+TM+) showed the highest prevalence of low socioeconomic status (*p* = 0.005) and the most severe cognitive deficits across all assessed domains (all *p* < 0.001). Cognitive reserve also differed significantly across groups (*p* = 0.004), with the ModCI+TM + group showing the lowest levels. Comorbid groups also carried the highest burden of depression and anxiety symptoms (both *p* < 0.001), with the highest rates of clinical cases. Subjective distress also varied significantly (*p* < 0.001). Both the physical (*p* = 0.019) and mental (*p* < 0.001) components of HRQoL showed progressive declines from controls to the most impaired comorbid group. No group differences were found in family support, HVLT-R Trial 1, Digit Span Forward, cortisol, or any exploratory synaptic protein (all *p* > 0.05) (Table 10).

**Table 10:** Biomarker comparisons across cognitive-psychiatric groups.

Biomarker	*p*-value
Inflammatory	
Neutrophil-to-lymphocyte ratio (NLR)	0.085
Lymphocyte-to-monocyte ratio (LMR)	0.121

Hormonal	*p*-value
Cortisol (ng/mL)	0.785
Synaptic proteins	
SYP (ng/mL)	0.942
SYT1 (ng/mL)	0.232
SYN2 (ng/mL)	0.621
Total protein (μg/mL)	0.196

### 3.4 Diagnosis-specific profiles within MCI

When the analysis focused on the subset of participants with mild cognitive impairment (*n* = 45), exploratory comparisons were performed across diagnostic groups (MCI-only, SZ + MCI, MDD + MCI, SUD + MCI) (Table 11). Given the small and unbalanced sample sizes, all results should be interpreted as exploratory and hypothesis-generating.

**Table 11:** Disorder-specific profiles among participants with MCI (*n* = 45).

Characteristic	MCI	SZ+MCI	MDD+MCI	SUD+MCI	*p*-value
Sociodemographic					
Socioeconomic level					0.019^1^
—Low	6 (33.3)*	4 (40.0)	5 (71.4)	9 (90.0)*	
—Medium	12 (66.7)*	6 (60.0)	2 (28.6)	1 (10.0)*	
Family support					0.048^1^
—Low	3 (16.7)*	1 (10.0)*	1 (14.3)	7 (70.0)*	
—Medium	4 (22.2)	3 (30.0)	2 (28.6)	1 (10.0)	
—High	11 (61.1)	6 (60.0)	4 (57.1)	2 (20.0)	
Cognitive test performance					
MoCA total score	28 [24.8–28.3]	26 [24–28]	25 [21–26]	25 [23–26]	0.038^2^
ITEM 1 (visuospatial ability)	5 [4.8–5]*	4 [3.75–5]	4 [3–5]	4 [3.8–4.3]*	0.024^2^
ITEM 5 (abstract reasoning)	18 (94.4)*	10 (50.0)*	7 (42.9)*	10 (50.0)*	0.015^1^
Stroop					
Word	12.5 [11–14]*	6 [3.5–8.3]*	9.5 [8–13]	9 [5–12.3]	0.002^2^
Color-word	13 [9.8–15]*	6.5 [5–10.3]*	10.5[7.8–12]	7.5 [5.8–8.3]*	<0.001^2^
Clinical characteristics					
Depression (HADS)					0.022^1^
—Normal	16 (88.9)*	3 (30.0)*	3 (42.9)	6 (60.0)	
—Possible case	2 (11.1)	3 (30.0)	1 (14.3)	3 (30.0)	
—Case	0 (0.0)	4 (40.0)	3 (42.9)	1 (10.0)	
Quality of life (SF-12)					
—Physical component	56.9 [45.7–60.9]	56.1 [46.3–59.2]	44.3 [36.6–55.6]	53.7 [40–59.5]	
—Mental component	48.7 [33.9–53.7]*	29.1 [24.7–43.7]	35 [20.6–50.6]	26.1 [20.3–40.5]*	0.020^2^
Molecular biomarkers					
Neutrophil-to-lymphocyte ratio (NLR)	1.05[0.88–1.47]	2.03[1.53–2.81]	1.61[1.03–2.14]	1.32[1.16–1.80]	0.007^2^
Synaptic proteins					
Synaptotagmin-1 (ng/mg)	0.36 [0.30–0.52]	0.31 [0.28–0.34]*	0.33 [0.26–0.48]	0.49 [0.33–0.67]*	0.029^2^

Data are presented as n (%) or median [IQR]. Significant overall group differences were assessed using χ² tests (1) or Kruskal-Wallis tests (2). Asterisks (*) indicate groups contributing to statistically significant between-group differences.

In exploratory unadjusted comparisons, sociodemographic disparities were marked: the SUD + MCI group showed the highest rates of low socioeconomic status (88.9%) and low family support (77.8%). Regarding cognition, groups differed in global cognition (MoCA total, *p* = 0.038) and specific domains: visuospatial/executive abilities (MoCA Item 1, *p* = 0.024), abstract reasoning (MoCA Item 5, *p* = 0.015), and inhibitory control (Stroop Word, *p* = 0.002; Stroop Color-Word, *p* < 0.001) (Table 11).

Depression severity also differed significantly across groups (*p* = 0.022), with SZ + MCI and MDD + MCI showing the highest rates of clinical cases. Mental HRQoL was lowest in the SZ + MCI and SUD + MCI groups (*p* = 0.020). At the biomarker level, peripheral inflammation differed significantly, with the SZ + MCI group showing the highest median NLR (*p* = 0.007). SYT1 also differed across diagnostic groups (*p* = 0.029) in the overall comparison, driven by higher levels in SUD + MCI compared with MDD + MCI. This exploratory finding should be interpreted cautiously given the small subgroup sizes and multiple comparisons. No significant differences were found in verbal memory, working memory, cognitive reserve, anxiety, subjective distress, physical HRQoL, cortisol, total protein, or the remaining exploratory synaptic proteins (all *p* > 0.05) (Tables 11, 12; Figure 1).

**Table 12:** Biomarker comparisons across diagnostic groups within mild cognitive impairment (*n* = 45).

Biomarker	*p*-value
Inflammatory	
Lymphocyte-to-monocyte ratio (LMR)	0.106

Hormonal	*p*-value
Cortisol (ng/mL)	0.334
Synaptic proteins	
SYP (ng/mL)	0.158
SYT1 (ng/mL)	0.200
SYN2 (ng/mL)	0.116

Mann–Whitney U tests for all comparisons (CI+ vs. CI−). No significant differences observed (all *p* > 0.05).

**Figure 1:**
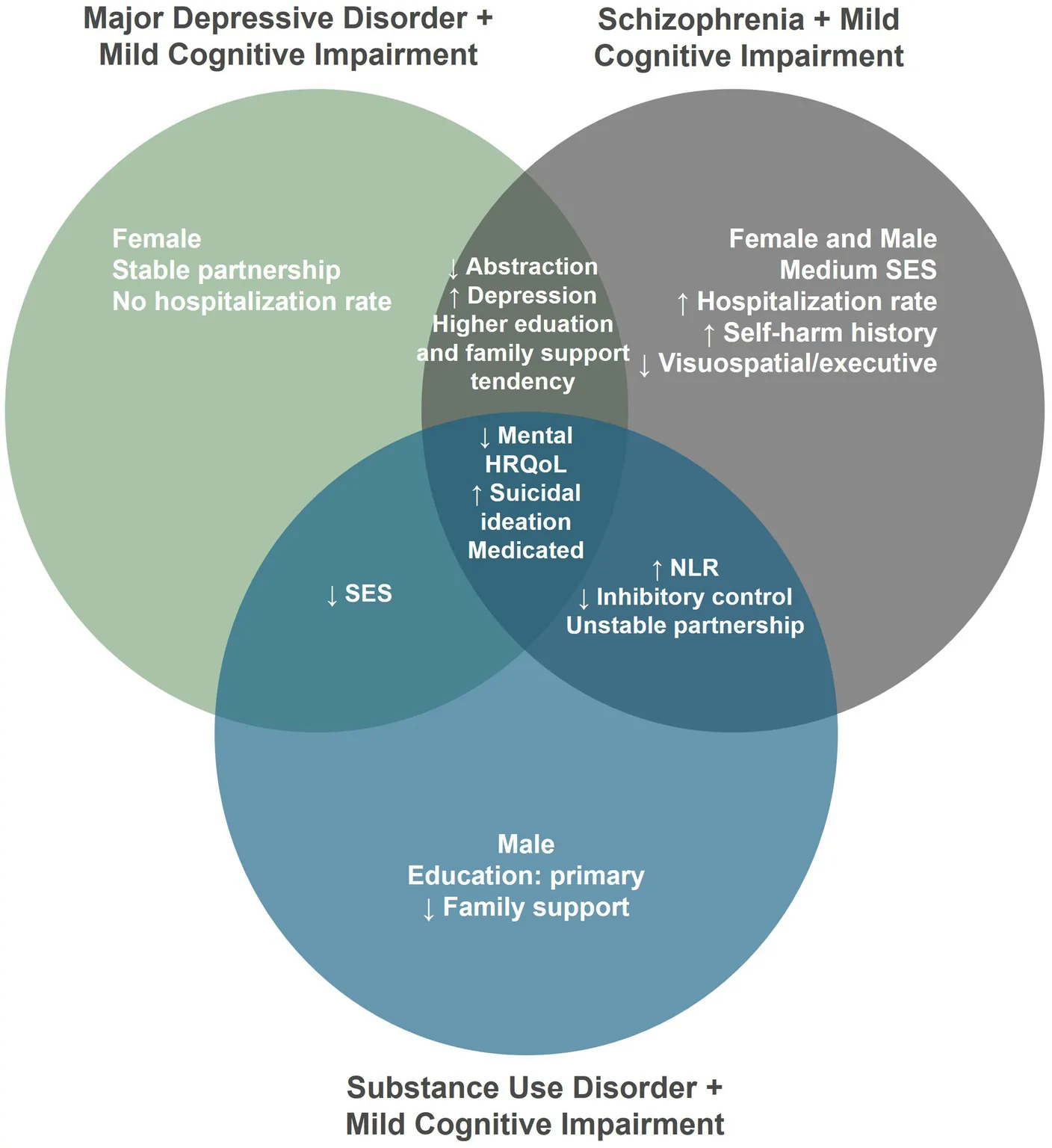
Exploratory Venn diagram showing diagnosis-specific and shared characteristics among participants with MCI.

### 3.5 Multivariate regression models

#### 3.5.1 Model 1: factors associated with cognitive impairment status

The primary logistic regression model was estimated in the complete-case biomarker subset (*n* = 91). Psychiatric disorder was independently associated with cognitive impairment [odds ratio (OR) = 5.23, 95% CI (2.05–13.35), *p* < 0.001]. SYP also remained in the final model after backward stepwise elimination and was independently associated with cognitive impairment (*B* = −0.007, SE = 0.003, OR = 0.993, 95% CI 0.986–0.999, *p* = 0.030). When expressed across the observed interquartile range, the corresponding OR was 0.681 (95% CI 0.481–0.964). All other candidate factors (including SYT1, SYN2, NLR/LMR, cortisol, cognitive reserve, and psychosocial measures) were removed during model refinement (Table 13).

**Table 13:** Results of binomial logistic regression (Model 1, *n* = 91): associated factors of global cognitive impairment.

Factors	B (SE)	OR	95% CI for OR	*p*-value
Constant	−0.181 (0.353)	0.835	–	0.608
Presence of a psychiatric disorder	1.655 (0.478)	5.234	2.052–13.349	< 0.001
Synaptophysin	− 0.007 (0.003)	0.993	0.986–0.999	0.030

Model Summary: Nagelkerke *R*^2^ = 0.214, Hosmer-Lemeshow *p* = 0.697. Final model after backward stepwise retained Psychiatric Disorder Status and Synaptophysin, both significant. B, unstandardized coefficient; SE, standard error; CI, confidence interval. Non-significant factors from the initial full model are shown for transparency.

Sensitivity analyses are presented in Supplementary Table S1. The association between psychiatric morbidity and cognitive impairment remained directionally consistent after sociodemographic adjustment (OR = 3.92, 95% CI 1.45–10.60, *p* = 0.007). In contrast, the association between SYP and cognitive impairment was attenuated and no longer statistically significant when all participants with available SYP data were included or after sociodemographic adjustment, supporting its interpretation as an exploratory and hypothesis-generating finding.

#### 3.5.2 Model 2: factors associated with subjective distress

In the final multinomial regression model, SF-12 Physical Component (PCS) (OR = 0.84, *p* = 0.002) and SF-12 Mental Component (MCS) (OR = 0.77, *p* < 0.001) were the only variables independently associated with subjective distress. Neither psychiatric diagnosis nor biomarkers (NLR, cortisol, exploratory synaptic proteins) showed significant associations with subjective distress (Table 14).

**Table 14:** Results of multinomial logistic regression (Model 2): associated factors of perceived stress.

	Stress category	B (SE)	OR	95% CI for OR	*p*-value
Intercept	Mild	23.220 (6.080)	–	–	<0.001
SF-12 physical component	Mild	− 0.179 (0.058)	0.836	0.746–0.937	0.002
SF-12 mental component	Mild	− 0.261 (0.073)	0.770	0.668–0.888	<0.001
Intercept	Moderate	32.109 (6.451)	–	–	<0.001
SF-12 physical component	Moderate	− 0.264 (0.064)	0.768	0.677–0.871	<0.001
SF-12 mental component	Moderate	− 0.393 (0.078)	0.675	0.580–0.786	<0.001
Intercept	Severe	35.708 (6.759)	–	–	<0.001
SF-12 physical component	Severe	−0.305 (0.071)	0.737	0.641–0.848	<0.001
SF-12 mental component	Severe	−0.524 (0.096)	0.592	0.490–0.715	<0.001

Model fit: pseudo R^2^ (Cox and Snell) = 0.673; Pseudo R^2^ (Nagelkerke) = 0.734. Model converged after excluding cortisol due to limited data (final *n* = 109). Only significant factors shown. Diagnostic group not retained. B, unstandardized; SE, standard error; CI, confidence interval.

#### 3.5.3 Model 3: factors associated with systemic inflammation

In the linear regression model log(NLR), only psychiatric disorder status was significantly associated with NLR (*β* = 0.32, *p* = 0.004). Subjective distress showed a non-significant inverse trend (*β* = −0.18, *p* = 0.093). Cognitive impairment was not significantly associated with NLR (Table 15; Figure 2).

**Table 15:** Results of linear regression (Model 3): associated factors of systemic inflammation.

Factor	Unstandardized B (SE)	Standardized β	*p*-value	95% CI for B
Constant	1.779 (0.339)	–	<0.001	1.106–2.452
Presence of a psychiatric disorder	1.082 (0.368)	0.315	0.004	0.350–1.814
Stress	− 0.366 (0.215)	−0.182	0.093	− 0.793 – 0.062

Model Summary: *R*^2^ = 0.095, Adjusted *R*^2^ = 0.074, *F*(2, 88) = 4.622, *p* = 0.012. Dependent variable log: (NLR). After backward elimination, the final model retained Psychiatric Disorder Status and Stress; only Psychiatric Disorder Status was significant. B, unstandardized; SE, standard error; CI, confidence interval.

**Figure 2:**
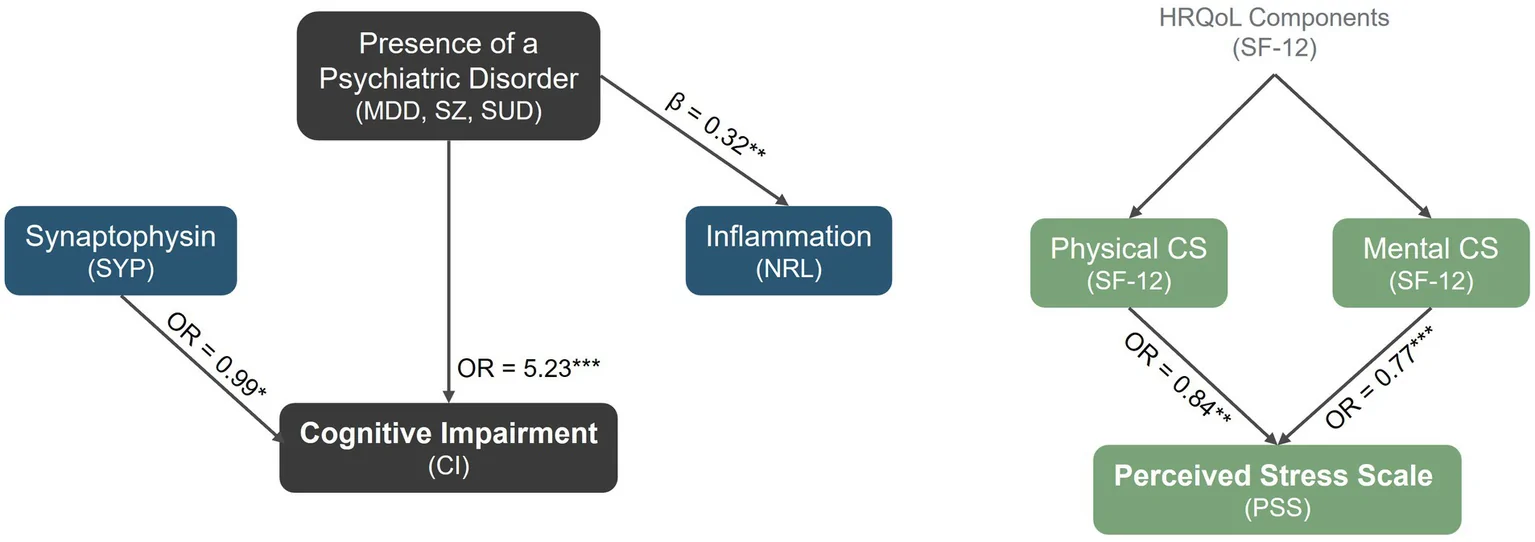
Transdiagnostic conceptual model summarizing independent factors of cognitive impairment, systemic inflammation, and subjective distress.

### 3.6 Analysis of sex differences

Significant sex differences were observed in three domains. Men showed higher cortisol levels and better perceived physical health, whereas women demonstrated superior verbal learning performance (all *p* < 0.05). No sex differences were found in inflammatory markers, other cognitive domains, mental HRQoL, or in exploratory salivary synaptic protein levels (all *p* > 0.05) (Tables 16, 17). Given the exploratory nature of sex-stratified analyses and the number of comparisons performed, these findings should be interpreted cautiously.

**Table 16:** Study variables showing significant sex differences.

Variable/domain	Men (*n* = 62)	Women (*n* = 69)	*p*-value
Neuroendocrine marker			
Cortisol levels	0.00 [0.00–2.10]	0.00 [0.00–0.00]	<0.001
Cognitive performance			
HVLT-R: total recall	8.0 [5.5–10.0]	9.5 [7.0–12.0]	0.020
Quality of life			
SF-12: physical health	55.5 [49.4–58.2]	50.7 [41.5–56.5]	0.012

Results from Mann–Whitney U tests. Only variables with *p* < 0.05 are shown. Cortisol values of 0.00 ng/mL indicate concentrations below the assay’s detection limit (0.93 ng/mL).

**Table 17:** Sex differences in synaptic protein levels.

Biomarker	Sample size (men/women)	Mann–Whitney U test	Standardized test statistic (Z)	*p*-value
SYP	*n* = 129 (60/69)	1524.00	0.072	0.943
SYT1	*n* = 110 (54/56)	2473.50	1.869	0.062
SYN2	*n* = 130 (61/69)	2320.50	0.991	0.322
Total protein (μg/mL)	*n* = 131 (69/62)	2189.00	0.230	0.818

Mann–Whitney U tests are compared. No significant differences (all *p* > 0.05). Sample sizes vary due to assay volume requirements.

## 4 Discussion

This transdiagnostic analysis provides evidence that the presence of a psychiatric disorder was the strongest independent correlate of global cognitive impairment, conferring more than a five-fold increase in odds (OR = 5.23). Systemic inflammation was also elevated among individuals with psychiatric disorders (NLR: *β* = 0.32), although it was not independently associated with cognitive impairment. In multivariable models, SYP remained associated with lower odds of cognitive impairment in the primary complete-case biomarker model (OR = 0.993). However, this association was attenuated in sensitivity analyses and should therefore be considered exploratory and hypothesis-generating pending independent replication.

In contrast, subjective distress was not linked to diagnosis, inflammation, or biomarkers. Instead, physical and mental HRQoL were the only psychosocial variables independently associated with subjective distress, with the model explaining a substantial proportion of the variance (Nagelkerke *R*^2^ = 0.734). These findings indicate a close relationship between HRQoL and subjective distress in this sample. Given the cross-sectional design and the use of stepwise procedures, these associations should be interpreted as associational rather than predictive.

Overall, the results are consistent with two partially dissociable patterns of association: (a) a biological–cognitive pattern centered on psychiatric morbidity, with an exploratory association involving SYP and a related inflammatory correlate (NLR), and (b) a psychosocial–subjective dimension characterized by HRQoL and subjective distress.

### 4.1 Beyond diagnostic categories: implications for dimensional frameworks

The finding that, in our primary analyses, the presence of any psychiatric disorder is a stronger factor associated with cognitive impairment than specific diagnostic labels provides direct empirical support for a dimensional perspective of psychopathology. This result is supported by meta-analytic evidence confirming substantial, shared neurocognitive deficits across a wide range of psychiatric disorders (East-Richard et al., 2020), the high rates of cognitive impairment observed early in the course of the disorder (Reviriego et al., 2025; Stainton et al., 2023), and its persistence during remission (Czerwińska and Pawłowski, 2020; Neu et al., 2019).

This pattern of transdiagnostic cognitive risk is consistent with contemporary theoretical frameworks, including the concept of a cognitive “Factor C” (Abramovitch et al., 2021) and the Hierarchical Taxonomy of Psychopathology (HiTOP), which conceptualizes psychopathology, including cognitive impairment, as a continuous, cross-diagnostic spectrum that transcends categorical boundaries (Kotov et al., 2017). This does not negate the clinical value of traditional diagnoses or the possibility of diagnosis-related differences in cognitive profiles but highlights that a substantial proportion of cognitive vulnerability is shared across disorders, underscoring the need for assessment and intervention strategies that extend beyond categorical boundaries.

### 4.2 Integrating peripheral inflammation and cognitive impairment

Psychiatric disorder status was independently associated with elevated NLR, indicating a reproducible link between psychiatric illness and low-grade systemic inflammation. Although we replicated the association between psychiatric status and elevated systemic inflammation, indexed by NLR (Canli, 2024; Chen et al., 2025), this marker did not independently predict cognitive impairment, suggesting that inflammation may reflect state-related psychiatric morbidity rather than being a proximal correlate of cognitive deficits. Taken together, these observations imply that peripheral inflammation functions as a biological correlate of psychiatric morbidity rather than a direct mechanistic contributor to cognitive impairment. These findings are consistent with recent evidence suggesting that inflammatory markers may track psychiatric illness burden more closely than cognitive status itself.

### 4.3 Salivary synaptic proteins: technical feasibility informs the path for future biomarker research

This study demonstrates that exploratory synaptic proteins (SYP, SYT1, SYN2) can be reliably quantified in saliva from a psychiatric sample (CV < 10%), supporting the technical feasibility of minimally invasive biomarker assessment. This technical success supports further investigation of salivary synaptic proteins in psychiatric populations and provides a basis for future validation studies. In multivariable analyses, SYP showed a modest independent association with cognitive status, whereas SYT1 and SYN2 were not retained. As the odds ratio was expressed per ng/mL increase, its magnitude should be interpreted in relation to the scale and distribution of the exploratory biomarker. When expressed across the observed interquartile range, the corresponding OR was 0.681 (95% CI 0.481–0.964), providing a more clinically interpretable estimate of the association, although the finding remains exploratory. Given the effect size and the absence of univariate group differences, SYP should be interpreted as a tentative and exploratory peripheral indicator related to cognitive status rather than as a validated diagnostic marker. Importantly, the present findings do not support the use of salivary synaptic proteins as diagnostic or screening tools in their current form. Similarly, recent systematic reviews highlight that no psychiatric biomarker currently achieves sufficient accuracy for routine clinical implementation, underscoring the need for longitudinal and multimodal validation approaches (Ricci et al., 2026).

This distinction refines the emerging literature on synaptic proteins as potential blood-based markers of cognitive decline (Pan et al., 2025). Although synaptic dysfunction is clearly implicated in cognitive impairment (Chang et al., 2021), our findings suggest that salivary synaptic proteins appear technically promising but require rigorous validation to establish biological origin and clinical relevance. Priority next steps include multimodal validation to clarify whether salivary synaptic proteins reflect central synaptic biology (Chang et al., 2021) and longitudinal designs to determine whether they track cognitive trajectories or psychiatric illness states.

### 4.4 Heterogeneity within cognitive impairment: clues from cognitive profiles

Although our findings support transdiagnostic cognitive risk, exploratory comparisons across cognitively impaired subgroups revealed preliminary heterogeneity. Participants with SZ (SZ + MCI) and MDD (MDD + MCI) exhibited more pronounced deficits in abstract reasoning, whereas the SUD (SUD + MCI) group showed more pronounced inhibitory-control impairments. Biologically, the SZ + MCI group exhibited the highest NLR, aligning with studies linking this inflammatory marker to cognitive severity in SZ (Chen et al., 2025; Syahna et al., 2025). Additionally, SYT1 levels varied across diagnostic groups, with higher levels in the SUD + MCI group than in MDD + MCI, though these comparisons are hypothesis-generating because of the small subgroup sizes and require replication in larger samples.

This pattern is compatible with dual-level dimensional models such as HiTOP, which propose a broad shared liability for cognitive impairment upon which additional diagnosis-specific features may be superimposed (Kotov et al., 2017). Accordingly, our findings suggest that psychiatric morbidity may be more closely related to the overall presence of cognitive impairment, whereas diagnosis may contribute to variability in its specific cognitive, biological, and psychosocial profile. All diagnosis-specific patterns remain exploratory and preliminary due to the small and unbalanced subgroup sizes (*n* = 7–10) and require replication in larger samples. Importantly, these exploratory diagnosis-specific findings do not contradict the primary transdiagnostic result but rather complement it by suggesting that psychiatric morbidity may be associated with the presence of cognitive impairment, whereas diagnostic category may contribute to heterogeneity once cognitive dysfunction is present.

### 4.5 Health-related quality of life as a transdiagnostic and functional dimension

In the present study, perceived stress was conceptualized as a marker of subjective distress, allowing the analytical focus to shift toward HRQoL as a broader functional construct. This suggests a distinction across domains: whereas objective cognitive impairment aligns with psychiatric morbidity and biological correlates, subjective distress appears more closely associated with perceived physical and mental HRQoL. From a clinical perspective, this pattern suggests that HRQoL may represent an important target for future longitudinal and interventional research aimed at understanding and potentially reducing subjective distress across diagnostic categories.

An alternative interpretation should also be considered. The strong association between subjective distress and the SF-12 components, particularly the mental component, may partly reflect conceptual overlap between self-reported measures of perceived stress and perceived HRQoL. Both instruments assess subjective experiences of wellbeing and psychological functioning, which may contribute to the high proportion of explained variance observed in the model. Nevertheless, the absence of associations with diagnosis, inflammatory markers, and salivary biomarkers suggests that subjective distress in this sample was more closely linked to perceived functioning and wellbeing than to the biological variables examined.

HRQoL is a multidimensional construct encompassing physical, psychological, and social wellbeing, which in recent decades has increasingly been recognized as a key outcome indicator in mental health research. Beyond symptom severity, HRQoL captures both the functional and subjective impact of mental disorders on individuals’ daily lives, as well as their clinical trajectories over time. In this regard, HRQoL can be conceptualized as a transdiagnostic factor, as it is consistently affected across different mental disorders and reflects shared processes that transcend specific diagnostic categories (Arango et al., 2021; Verdejo-Garcia et al., 2024).

In line with previous evidence, cognitive impairment has been identified as a major factor associated with HRQoL across psychiatric disorders. In MDD, cognitive deficits are strongly associated with poorer functional recovery and reduced HRQoL. Meta-analytic evidence indicates that interventions targeting cognitive impairment in patients with depression are associated with improved overall clinical outcomes, highlighting cognition as a key therapeutic target beyond mood symptom improvement (Rhee et al., 2024). Similarly, in SZ, cognitive impairment is consistently associated with poorer psychosocial functioning and HRQoL, and cognitive remediation interventions have demonstrated benefits on functional outcomes (Martini et al., 2024). Moreover, depressive symptoms and cognitive deficits independently contribute to poorer HRQoL in individuals with SZ, suggesting a cumulative negative impact on everyday functioning (Pascal de Raykeer et al., 2019). In SUDs, HRQoL similarly represents a key indicator of functional and clinical burden. Individuals with SUDs, particularly those with comorbid severe mental disorders, consistently report poorer HRQoL across multiple domains compared with the general population (Adan et al., 2017). Cognitive factors such as impaired inhibitory control, together with substance use patterns and psychosocial vulnerability, have been linked to reduced HRQoL in this population (Fatima et al., 2024).

From a transdiagnostic perspective, recent evidence further supports models that conceptualize HRQoL as closely linked to shared cognitive and functional processes across mental disorders. Profiles characterized by alterations in cognitive control and motivational systems are associated with poorer functioning and reduced HRQoL regardless of diagnostic labels (Verdejo-Garcia et al., 2024). Similarly, large-scale evidence-based syntheses have emphasized the relevance of functional, psychosocial, and contextual factors in shaping the development and course of mental disorders across diagnoses (Arango et al., 2021). Together with our results, this body of evidence supports integrative clinical models in which biological, cognitive, and psychosocial dimensions are considered simultaneously rather than hierarchically.

Emerging longitudinal research suggests that HRQoL, particularly its mental component, may index broader cognitive vulnerability. Higher HRQoL has been associated with a lower risk of subsequent cognitive impairment and dementia (Phyo et al., 2021). Sex-specific patterns have been reported, with women tending to report poorer HRQoL across multiple domains (Oliveira et al., 2025), highlighting the importance of considering sociodemographic factors and adopting a gender-sensitive perspective in future research. These observations further highlight the relevance of HRQoL as an early and clinically meaningful indicator of vulnerability, complementing biological and cognitive assessments.

Overall, the available evidence, together with our findings, supports an integrative model in which cognitive impairment and HRQoL represent interrelated but partially independent transdiagnostic dimensions, differing in their biological and psychosocial correlates yet jointly shaping functional outcomes and recovery in psychiatric disorders. Both dimensions are likely to be important for clinical course, functioning, and recovery in individuals with mental disorders (Figure 3).

**Figure 3:**
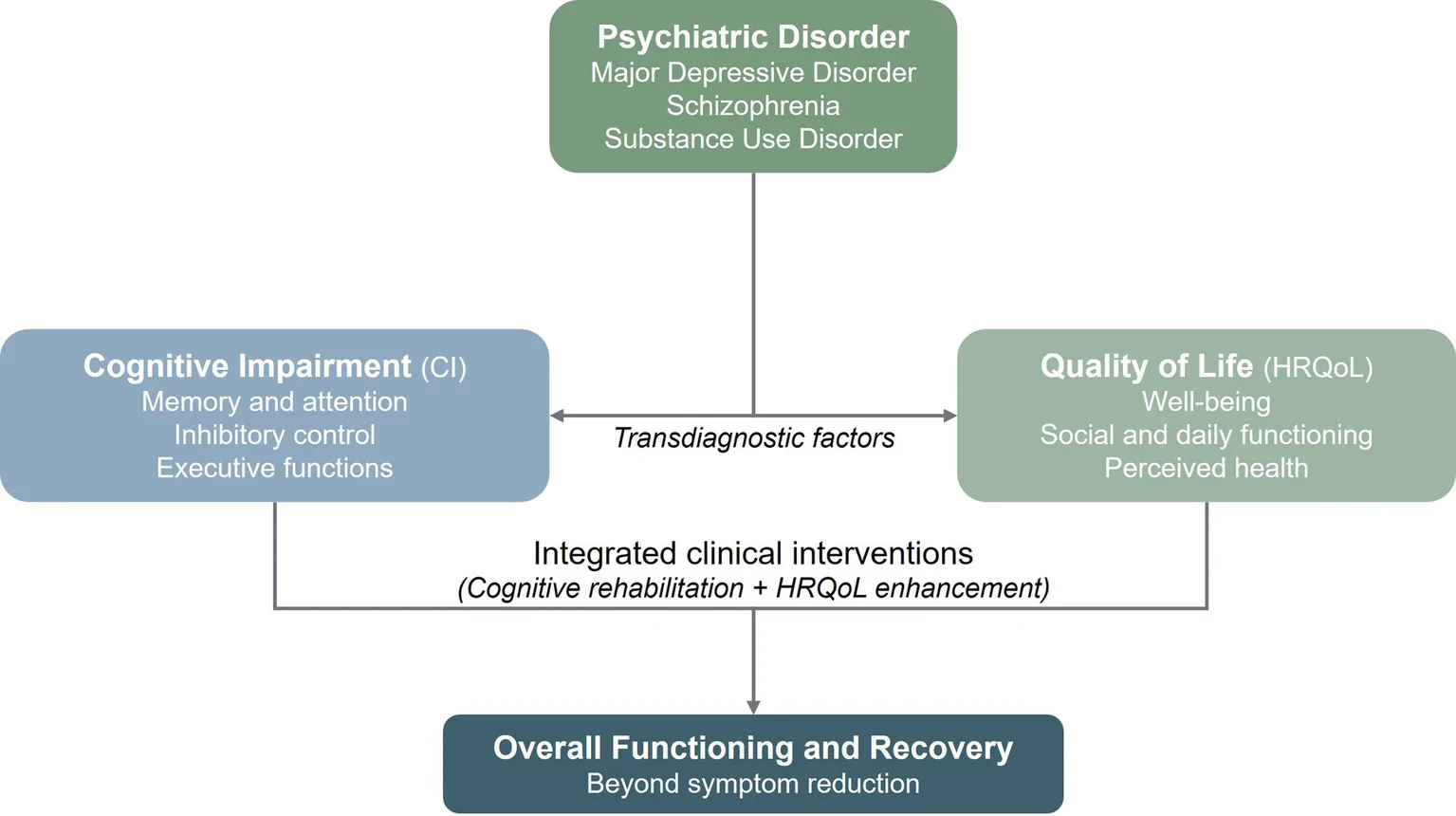
Integrated model of cognition, HRQoL, and functional outcomes in psychiatric disorders.

### 4.6 Limitations

Several limitations must be acknowledged. First, the cross-sectional design precludes causal inference and does not allow conclusions regarding the temporal direction of the observed associations. Second, although adequate for the primary transdiagnostic analyses, the sample size provides limited statistical power for diagnostic subgroup comparisons, which should therefore be interpreted as exploratory. Recruiting clinically stable psychiatric patients who also meet strict criteria for cognitive and biomarker assessment is inherently difficult, which contributed to the unbalanced group sizes. Third, the neuropsychological assessor was not blinded to participants’ diagnostic status. However, this limitation was mitigated through standardized test administration and norm-referenced scoring. Fourth, universal psychopharmacological treatment precludes disentangling illness from medication effects, a critical confounder in psychiatric research. Fifth, the use of non-standardized clinical blood draws for NLR, although pragmatic, introduces measurement noise due to variability in blood draw timing (e.g., fasting state, circadian rhythm). Sixth, variable saliva yield led to differing sample sizes across salivary biomarkers, an expected constraint in psychiatric samples due to medication-induced xerostomia. Biomarker missingness also differed across some diagnostic groups, particularly for NLR and SYP, which may have influenced complete-case biomarker analyses despite the sensitivity analyses performed (Supplementary Tables S1, S2). Finally, because salivary synaptic proteins represent a novel biomarker modality, their biological origin (central vs. peripheral) and relation to brain synaptic integrity remain to be clarified. Multimodal and longitudinal validation will be required.

The present manuscript reports the baseline cross-sectional assessment of an ongoing prospective longitudinal cohort study. Longitudinal analyses aimed at examining the temporal evolution of cognitive, biological, and psychosocial variables are currently underway and will be reported separately.

## 5 Clinical implications, future directions, and conclusion

First, the strong transdiagnostic association between psychiatric morbidity and cognitive impairment supports the routine use of brief cognitive screening across psychiatric settings, regardless of diagnosis. Second, the observed associations between psychiatric morbidity, cognitive impairment, inflammation, and HRQoL support the development of future longitudinal and intervention studies aimed at clarifying their temporal and potentially modifiable relationships. In particular, further research should examine whether improvements in psychiatric status, inflammatory burden, or HRQoL are associated with changes in cognitive and subjective outcomes over time. Third, although salivary synaptic proteins did not demonstrate stand-alone clinical utility in this cross-sectional study, the small exploratory association observed for SYP supports further investigation as a minimally invasive research biomarker, pending independent replication and rigorous validation (Figure 4).

**Figure 4:**
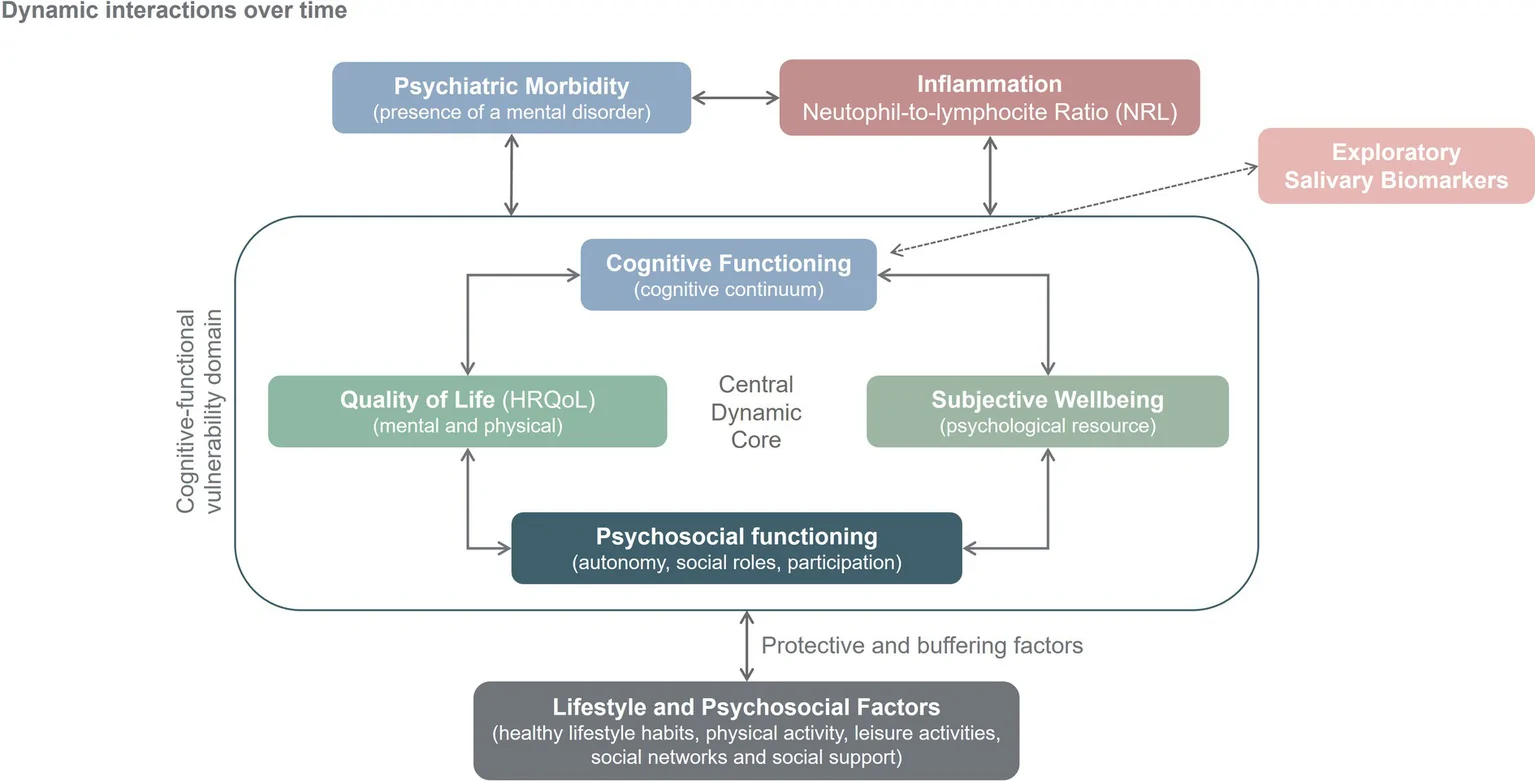
Beyond diagnostic labels: a transdiagnostic baseline characterization of cognitive functioning, inflammation, and HRQoL in a prospective psychiatric cohort.

In conclusion, this baseline study advances a multilevel, dimensional framework for understanding cognitive impairment in psychiatry. The presence of psychiatric morbidity emerged as the primary transdiagnostic correlate of cognitive impairment, whereas exploratory analyses suggested that diagnostic category may contribute to heterogeneity in cognitive, biological, and psychosocial profiles. A modest exploratory association with salivary synaptophysin was also observed, although its biological significance and clinical relevance remain uncertain and warrant independent replication. These findings support hierarchical models in which a shared liability underlies cognitive dysfunction across disorders, with diagnosis-specific biological and psychosocial features that may be superimposed. At the same time, HRQoL emerged as a distinct dimension strongly associated with subjective distress. This dissociation highlights complementary but partially independent biological-cognitive and psychosocial-subjective dimensions of psychiatric burden, both of which are meaningfully related to functional outcomes and recovery.

Importantly, the present work demonstrates the technical feasibility of quantifying exploratory salivary synaptic proteins in psychiatric populations, representing a necessary first step toward minimally invasive biomarker development, while underscoring that longitudinal validation and confirmation of biological origin remain essential. By situating these findings within an explicitly longitudinal framework, this baseline study provides a reference point for future investigations aimed at delineating trajectories of cognitive and functional vulnerability in real-world psychiatric care.

## Data Availability

The raw data supporting the conclusions of this article will be made available by the authors, without undue reservation.
