# Supplementary material for: Psychiatric morbidity is associated with cognitive impairment, whereas diagnostic category may shape its profile: a transdiagnostic study of inflammation, salivary biomarkers, and health-related quality of life
Source: Front Psychol. 2026 Oct 1;17:1913919. doi: 10.3389/fpsyg.2026.1913919 (PMC13669180; doi:10.3389/fpsyg.2026.1913919)
Supplement: Supplementary file 1 [file Table_1.DOCX]

**Supplementary Table S1. Sensitivity Analyses of the Primary Logistic Regression Model for Cognitive Impairment**

| **Model** | **n** | **Psychiatric morbidity OR (95% CI)** | **p-value** | **SYP OR (95% CI)** | **p-value** |
| --- | --- | --- | --- | --- | --- |
| Primary complete-case biomarker model | 91 | 5.23 (2.05-13.35) | <0.001 | 0.993 (0.986-0.999) | 0.030 |
| All available SYP cases | 109 | 4.61 (1.99-10.69) | <0.001 | 0.994 (0.988-1.000) | 0.069 |
| Sociodemographic-adjusted model | 109 | 3.92 (1.45-10.60) | 0.007 | 0.995 (0.989-1.001) | 0.105 |
| Expanded psychosocial-adjusted model | 109 | 2.97 (0.86-10.27) | 0.085 | 0.993 (0.986-1.001) | 0.092 |
| Medication-adjusted model | 109 | 2.25 (0.53-9.57) | 0.274 | 0.996 (0.989-1.002) | 0.179 |

Primary model corresponds to the complete-case biomarker subset used in the main regression analysis. Sociodemographic-adjusted model included age, sex, years of education, socioeconomic status, and family support. The expanded model additionally included cognitive reserve, anxiety, depression, and SF-12 physical and mental component scores. The medication-adjusted model additionally included current psychopharmacological treatment burden. SYP = synaptophysin
