# Supplementary material for: Psychiatric morbidity is associated with cognitive impairment, whereas diagnostic category may shape its profile: a transdiagnostic study of inflammation, salivary biomarkers, and health-related quality of life
Source: Front Psychol. 2026 Oct 1;17:1913919. doi: 10.3389/fpsyg.2026.1913919 (PMC13669180; doi:10.3389/fpsyg.2026.1913919)
Supplement: Supplementary file 2 [file Table_2.DOCX]

**Supplementary Table S2. Analysis of Biomarker Missingness**

| **Biomarker** | **Available n** | **Missing n** | **Cognitive impairment status** | **Psychiatric morbidity** | **Diagnostic group** |
| --- | --- | --- | --- | --- | --- |
| NLR | 106 | 25 | p = 0.381 | p = 0.044 | p = 0.028 |
| Cortisol | 69 | 62 | p = 0.728 | p = 0.221 | p = 0.300 |
| SYP | 110 | 21 | p = 1.000 | p = 0.059 | p = 0.008 |
| SYT1 | 129 | 2 | p = 1.000 | p = 1.000 | p = 0.500 |
| SYN2 | 130 | 1 | p = 1.000 | p = 0.481 | p = 1.000 |

P-values compare participants with available versus missing biomarker observations. Missingness did not differ according to cognitive impairment status for any biomarker. NLR and SYP showed differential missingness across diagnostic groups.
