# Supplementary material for: Psychiatric morbidity is associated with cognitive impairment, whereas diagnostic category may shape its profile: a transdiagnostic study of inflammation, salivary biomarkers, and health-related quality of life
Source: Front Psychol. 2026 Oct 1;17:1913919. doi: 10.3389/fpsyg.2026.1913919 (PMC13669180; doi:10.3389/fpsyg.2026.1913919)
Supplement: Supplementary file 3 [file Table_3.DOCX]

**Supplementary Table S3. Age and years of education across diagnostic groups.**

| **Variable** | **Controls (n=63)** | **MDD (n=20)** | **SZ (n=20)** | **SUD (n=28)** | **p-value** |
| --- | --- | --- | --- | --- | --- |
| Age (years) | 32.0 [27.0-63.5] | 54.0 [45.0-63.2] | 45.5 [34.2-48.0] | 46.5 [43.8-53.2] | 0.028 |
| Years of education | 17.0 [15.0-19.0] | 14.5 [12.0-16.0] | 12.5 [10.0-14.0] | 9.5 [6.0-12.0] | <0.001 |

Values are presented as median [interquartile range]. P-values were obtained using Kruskal-Wallis tests. Post-hoc Benjamini-Hochberg-adjusted comparisons showed that years of education were significantly higher in controls compared with MDD, SZ, and SUD groups, and significantly lower in SUD compared with MDD and SZ groups. For age, only the MDD versus SZ comparison remained significant after adjustment.
