# Supplementary material for: Psychiatric morbidity is associated with cognitive impairment, whereas diagnostic category may shape its profile: a transdiagnostic study of inflammation, salivary biomarkers, and health-related quality of life
Source: Front Psychol. 2026 Oct 1;17:1913919. doi: 10.3389/fpsyg.2026.1913919 (PMC13669180; doi:10.3389/fpsyg.2026.1913919)
Supplement: Supplementary file 4 [file Table_4.DOCX]

**Supplementary Table S4. Distribution of Cognitive Impairment Severity Across Diagnostic Groups**

| **Diagnostic Group** | **No Impairment** | **MCI** | **Moderate Impairment** | **Severe Impairment** | **Missing** | **Total** |
| --- | --- | --- | --- | --- | --- | --- |
| Controls | 41 | 18 | 4 | 0 | 0 | 63 |
| MDD | 10 | 7 | 2 | 1 | 0 | 20 |
| SZ | 3 | 10 | 4 | 3 | 0 | 20 |
| SUD | 8 | 10 | 7 | 2 | 1 | 28 |
| Total | 62 | 45 | 17 | 6 | 1 | 131 |

One participant in the SUD group did not complete the MoCA assessment and therefore could not be classified according to cognitive impairment status.
